# Adaptation of diabetes prevention program for Chinese Americans – a qualitative study

**DOI:** 10.1186/s12889-022-13733-5

**Published:** 2022-07-11

**Authors:** Ming-Chin Yeh, Wincy Lau, Siqian Chen, Ada Wong, Ho-Jui Tung, Grace X. Ma, Judith Wylie-Rosett

**Affiliations:** 1 Nutrition Program, School of Urban Public Health, Hunter College, City University of New York, New York, NY USA; 2 Chinese Community Partnership for Health, New York Presbyterian-Lower Manhattan Hospital, New York, NY USA; 3 Department of Health Policy and Community Health, Jiann-Ping Hsu College of Public Health, Georgia Southern University, Statesboro, GA USA; 4 Center for Asian Health, Lewis Katz School of Medicine, Temple University, Philadelphia, PA USA; 5 Department of Epidemiology and Population Health, Albert Einstein College of Medicine, Bronx, NY USA

**Keywords:** Chinese Americans, Web-based diabetes prevention program, Cultural and linguistic adaptation, Focus groups, Lifestyle intervention, Qualitative study

## Abstract

**Background:**

Studies have demonstrated that a culturally and linguistically tailored Diabetes Prevention Program (DPP) can be effective in reducing diabetes risk in Chinese Americans. The purpose of this study was to explore the cultural and linguistic acceptability of the Centers for Disease Control and Prevention’s Prevent T2 curriculum in an online format in the Chinese American community in New York City (NYC).

**Methods:**

Three focus groups among a total of 24 Chinese Americans with prediabetes and one community advisory board (CAB) meeting with 10 key stakeholders with expertise in diabetes care and lifestyle interventions were conducted. Each focus group lasted approximately 1 to 1.5 h. All groups were moderated by a bilingual moderator in Chinese. The sessions were audiotaped, transcribed and translated to English for analysis. Using Atlas.ti software and open coding techniques, two researchers analyzed transcripts for thematic analysis.

**Results:**

Five key themes were identified: barriers to behavioral changes, feedback on curriculum content and suggestions, web-based intervention acceptability, web-based intervention feasibility, and web-based intervention implementation and modifications. Participants with prediabetes were found to have high acceptability of web-based DPP interventions. Suggestions for the curriculum included incorporating Chinese American cultural foods and replacing photos of non-Asians with photos of Asians. Barriers included lack of access to the internet, different learning styles and low technology self-efficacy for older adults.

**Conclusion:**

Although the acceptability of web-based DPP in the Chinese American community in NYC is high, our focus group findings indicated that the major concern is lack of internet access and technical support. Providing support, such as creating an orientation manual for easy online program access for future participants, is important.

## Introduction

Diabetes is one of the most common diseases in the United States, with an overall prevalence of 34.2 million people, representing 10.5% of the US population [1, 2]. In New York City (NYC) alone, more than 1 million people have diabetes, 17.7% of whom are Non-Hispanic Asians, according to the Centers for Disease Control and Prevention (CDC) [2]. Furthermore, among the various Asian ethnic groups in NYC, the Asian American Federation estimates that the Chinese population is the largest [3]. The number of Chinese Americans in NYC expected to grow as the entire US Asian population is projected to reach 46 million by 2060, compared to 22 million in 2019, according to Pew Research Center [4].

Health disparities among Asian New Yorkers showed that the prevalence of diabetes among Chinese Americans in NYC is 9.1%, while one in three Chinese American adults did not exercise in the past 30 days, which increases the risk of diabetes [5]. Studies also showed that Chinese American immigrants have a higher risk of diabetes than those who live in China [6]. The diabetes management behaviors in Chinese Americans are poorer than Black and Hispanic Americans, and they are less likely to receive recommended physician-led management practices and are less likely to engage in diabetes self-management practices [7].

The steep increase in type 2 diabetes cases, as well as its complications and health expenditures became a significant public health issue in the United States [6]. According to the American Diabetes Association, the estimated total cost of diagnosed diabetes rose from $245 to $327, with a 26% increase over five years from 2012 to 2017 [8]. People with diabetes have 2.3 times higher medical expenditures than those who do not have diabetes [8]. Diabetes is a common cause of hospitalization and accounted for three-quarters of the cost of diabetes hospitalizations paid by Medicare and Medicaid in 2003 [9]. Therefore, there is an urgent need to implement programs that help people prevent and manage diabetes.

The Diabetes Prevention Program (DPP) was initially developed in 1996 as a three-year randomized controlled clinical that focuses on healthy eating and physical activity for people with prediabetes to examine the effectiveness of reducing their risk of type 2 diabetes [10]. The national DPP was authorized in 2010 by Congress because of the potential benefits of preventing diabetes [11]. Studies showed that the DPP intervention leads to a more significant reduction in the incidence of type 2 diabetes than using medications, and it continued to show a lower incidence of type 2 diabetes at five years of follow-up [12]. The implementation of the DPP curriculum to participants at age 50 can prevent 37% of new cases of type 2 diabetes by age 65 and lower the cost of medical expenses [13].

In addition, the online platform has been shown to serve as an effective way to implement the DPP program as digital technology is growing [13]. For example, an online DPP study demonstrated improved hemoglobin A1c (HbA1c) and weight reduction among participants after completing the online lessons [13]. A study conducted among older adults indicated that the online DPP could help them to achieve weight loss and had a higher completion rate compared to in-person sessions [14]. Similarly, a meta-analysis showed that web-based interventions had better improvement in both knowledge and desired behavioral outcomes compared to non-web-based interventions [15].

There were, however, limited studies conducted in the Chinese American community with a translated DPP curriculum. Culturally tailored interventions can improve healthcare equity and quality for minority populations and are effective in improving clinical and psycho-behavioral outcomes [16, 17]. However, a systematic review identified that the framework and guidelines for developing a culturally tailored intervention are often unclear. Therefore, community-based participatory research [18] is essential to elicit input to adapt the DPP and guide the development of an intervention to best address the needs of high risk Chinese Americans. Our 2016 study [19], and Wang et al [20] showed that the participants achieved weight loss and improved HbA1c concentration with a culturally and linguistically tailored DPP curriculum. After the publication of our previous study [19], the CDC updated its original 2002 Diabetes Prevention Program (DPP) and launched a new curriculum named PreventT2 in 2016. Thus, the purpose of this study was to conduct qualitative research using focus groups to 1) explore the cultural and linguistic acceptability of our revised PreventT2 curriculum, and 2) explore perceived feasibility and acceptability of transforming the PreventT2 curriculum into an online format from Chinese Americans living in NYC. We plan to incorporate the feedback received form the focus group discussion to fine-tune the revised PreventT2 curriculum before future interventions.

## Methods

### Study design

This qualitative study uses focus groups to collect participants’ comments/feedback on the translated PreventT2 curriculum. Three focus groups of community members plus one meeting of community advisory board (CAB) members were conducted. All focus group discussion took place at a community-based organization in Manhattan’s Chinatown in NYC. The reporting of this paper follows the principles of the Consolidated Criteria for Reporting Qualitative Research (COREQ) [21]. This study received Institutional Review Board approval from Hunter College, City University of New York.

### Participants

A purposive sample of study participants were recruited through community health fairs and physician referrals. The inclusion criteria for the focus groups included Chinese Americans with prediabetes [HbA1c 39–46 mmol/mol (5.7–6.4%)], body mass index (BMI) ≥ 23 kg/m^2^, no medical conditions for which the DPP lifestyle intervention would be contraindicated, speak Chinese, and willingness and ability to provide informed consent. A total of 24 participants were divided into three focus groups of eight people in each group. The focus groups were conducted between March and April 2019.

An additional focus group was conducted among the CAB members which represented a diverse group of leaders and key informants in the Chinese American community in NYC. The advisory board members have expertise in the areas of diabetes prevention and management, intervention design and program evaluation, and community outreach. In addition, two Chinese American students with nutrition backgrounds also participated. A total of 10 people participated in the CAB focus group, which was conducted in March 2020.

### Procedures

Focus group participants were asked to evaluate and provide feedback on the revised CDC PreventT2 curriculum. The curriculum is one-year in length and includes 16 weekly sessions in the core intervention followed by six monthly post-core interventions. A lifestyle coach would lead the discussion on topics such as healthy eating, physical activity, and stress reduction for participants with pre-diabetes. The goal of the program is to promote modest weight loss (5%-7%) through healthy lifestyle to prevent the onset of type 2 diabetes [22]. The curriculum was culturally and linguistically adapted to Chinese Americans by our team of researchers which includes members who are native speakers of Mandarin, Cantonese or both, with expertise and training in nutrition, diabetes prevention, and cultural competency [19].

During the focus group, a semi-structured discussion guide was used to facilitate the conversation (see Table 1 for focus group questions). A trained moderator (ZG) and the study principal investigator (MCY), who are both bilingual and of Chinese heritage, facilitated the groups in Chinese (Cantonese and/or Mandarin). Prior to the focus groups, participants were each given three to four chapters of the translated curriculum to provide feedback during the focus groups. Each group lasted approximately one hour, and the discussion was recorded for later transcription and translation to English. Similarly, a discussion guide was used in the CAB member group led by a bilingual moderator. The CAB group discussion lasted about 1.5 h and was again recorded, transcribed and translated to English for analysis. Transcripts, original and translated, were checked by MCY for accuracy to minimize potential error before analysis.

**Table 1:** Focus group discussion questions for the revised PreventT2 curriculum

A. Review of study materials
1. What do you think of materials for the program designed to reduce diabetes risk in Chinese immigrants? What do you like and what do you think needs to be changed? *[probe: too wordy/difficult to find information or easy to find information, *etc*. Topics that you think should or should not be included, too much or too little covered in each session.]* 2. What do you think about the format of the curriculum materials? What do you like or think needs to be changed about the way the printed pages look? Size and style of print, use of graphics and illustrations? *[probe: bound vs. loose leave handouts, *etc*.]* 3. How familiar are you with some of nutrition terms, such as cholesterol, saturated fatty acids, etc.? What information would be needed with respect to the definitions? What terms should we change so the terms will be more clear or easy to understand? 4. What else you want to let us know so that we can make the materials more appealing?
B. Intervention format to be delivered
1. First, have you participated in any online health promotion program before? a. If you have, what did you like about it? What didn't you like about it? b. If you have not, why not? 2. What do you think of delivering the DPP program online for Chinese immigrants? a. Will you like it? Why and why not? b. Do you think other people will like it? Why or why not? 3. If you think people will not like it, what do you think we should do to help make online delivery acceptable and successful?
C. Intervention components to be delivered
1. Are there anything you can think of to help people eat healthier? 2. Are there any fun activities that you can think of to help people exercise more? 3. What do you think we should do to help people stay motivated to eat healthy and be physically active? 4. In addition to the standard curriculum, we will offer a “tool-box” option to help you overcome some of your problem areas. What do you think we should offer that will be most helpful to you? *[probe: examples include cookbooks/recipes for healthy cooking, jump ropes for increasing physical activity, *etc*.]* 5. Self-monitoring or keeping track of what you eat and your physical activity is an important part of the curriculum as it helps monitor progress and promotes confidence for lifestyle changes a. What would be the ways we can offer to help participants keep daily records of their physical activity during the six-month core program? *[show a pedometer]* How can we make keeping track of physical activity easier? b. What would be the ways we can offer to help participants keep daily records of the food they eat during the core program? *[show a copy of the tracker]* What do you think are the advantages and disadvantages of keeping track daily? Gradually reducing the number of days a person keeps track of food intake? What can we do to make it more interesting and help people keep on with monitoring?

### Data analysis

Members of the research team (WL and SC), who are also bilingual (native Cantonese and Mandarin speakers) independently reviewed all transcripts of the three focus groups and one CAB meeting accuracy. The transcripts were analyzed thematically [23]. Analysis involved initial open coding of text, which was done by two team members (WL and SC) independently using Atlas.ti (version 8.0) [24] then repeatedly modified with the coders’ reading of each transcript together to resolve coding differences. If discrepancies in coding persisted, a third person (MCY) was invited to discuss the findings to seek agreement. The codes were then grouped into categories and generated emerging themes. An inductive approach was used to define the themes [23]. Reflexivity considerations such as cultural background, linguistic tradition, personal preferences, and social position were observed, especially during data collection and analysis, to reduce potential bias [25, 26]. In addition, aspects of social and cultural theories important to Chinese were used in the interpretation of findings [26].

## Results

Five key themes and 12 sub-themes were developed during the thematic analysis process. The five key themes were (1) Barriers to behavioral changes; (2) Feedback on curriculum content and suggestions; (3) Web-based intervention acceptability; (4) Web-based intervention feasibility; and (5) Web-based intervention implementation and modifications.

### Barriers to behavioral change

Internal and external barriers that prevented participants from making behavioral changes were identified. The internal barriers identified included comorbidities, pleasure in eating cultural foods and aging.

#### Internal barriers

Participants reported comorbidities as the major barriers from staying physically active. For example, one participant stated: *“I sometimes get out of breath when I walk because I have a weak body.”*

Several participants expressed preference for eating certain cultural staple foods as a barrier to make changes in their diet. Some perceived, albeit mistakenly, that being healthy is to stop eating rice and noodles and the foods they enjoy. For example, *“Chinese love rice. We need to change slowly. If I don’t eat rice at all, I won’t be used to it. I need a little rice” and “I grew up eating rice and noodles… and I was always hungry when I was young because of poverty… eating is very important you know?”.*

Some participants reported that aging limited people’s motivation and changing their lifestyle is not feasible. For example, *“Part of the older generations are thinking that they are old and food doesn’t matter to them when they have not many days left” and “My mother has diabetes. I told her not to overeat, and she is very unsatisfied with me and told me to just let her.”*

#### External barriers

Participants reported lack of family, medical and social support as having a negative impact on their ability to lead a healthy lifestyle. Most participants agreed that family support played an important role in sustaining their health behaviors in the context of household duties. A seemingly frustrated spouse commented: *“Sometimes my spouse doesn’t do anything, and I have to do everything. When I have these errands then I can’t cook, and I have nothing to eat when I’m hungry, so I eat bread and snacks. There’s nothing I can do. I eat snacks when I get home, and then I just sleep or watch TV.”*

In addition, participants reported a lack of support from healthcare professionals as hindering their motivation to change. *“…because usually doctors don’t tell you much. They will only say, move more and eat less. It is easy but can you do that? It’s not easy.”*

### Feedback on curriculum contents and suggestions

Participants provided valuable feedback for enhancing cultural and linguistic adaptations for the curriculum. Participants noted that the dietary habits and guidelines demonstrated in the curriculum were different from their usual diet. They suggested foods that could be included in the curriculum, such as bubble tea, milk tea and coffee, which are the most common beverages among Chinese Americans. They also noted that some foods in the original curriculum are not part of their usual diet and recommended the inclusion of Chinese cultural foods in the curriculum. For example, *“Americans will go to DD to buy a doughnut, but Chinese people will want to go to Da Ban Bakery to buy a piece of bread.”* One participant pointed out that Chinese Americans rarely have strawberries for breakfast as suggested in the DPP so they might not feel the diet is relevant to them. They also suggested the inclusion of common condiments such as soy sauce or oyster sauce instead of salsa or sour cream.

Importantly, both community members and CAB members pointed out that images in the curriculum should include Chinese foods and Asian faces to enhance familiarity. *“I think the pictures need to have Chinese or Asian faces instead of westerner faces” and “If I go online and see only westerner faces, I will lose interest because I think the class is not for us.”*

When participants were asked for examples of healthy lifestyle strategies that are relevant to Chinese populations, participants provided strategies on healthy cooking, nutrition, physical activity and stress relief methods. Most of them identified reducing fried and sugary food intake and increasing vegetable intake as a prime example of eating more healthily. The majority of participants liked to share meals with partners or divide the foods into several meals to reduce their overall intake. *“…I usually share one serving with my wife while our kid consumes another serving, so that our intake is reduced.”* Strategies to promote physical activities included standing up while watching TV and running with friends. Some also mentioned that exercising and singing could help them to reduce their stress.

### Web-based intervention acceptability

The online DPP was well-received, as the majority of participants were satisfied with the comprehensive nature of the program. Participants expressed that the curriculum would motivate them to make behavior changes and that they would promote this program to others. Participants also expressed that web-based programs would be very useful. For example, one participant said that he could read the curriculum while on public transportation using his phone: *“It is better for (online) sessions like this than to simply read books. In the long-term it is very beneficial.”*

In addition, participants were asked if they were interested in monitoring their diet and physical activities using smartphone applications. While some mentioned they were not familiar using applications on smartphones, some expressed interest as it was more convenient, and said they were receptive if instructions are provided. Participants also suggested photo sharing on social media as a method to promote diet monitoring as younger generations tend to take photos at mealtimes: *“Actually, this cell phone method is good…Younger people who are in their 20 s, 30 s, 40 s, love taking photos. They take photos for everything and share them on their social media to tell others what they eat…and they can eat and see each other’s photos. That will be good.”*

The acceptability of using a pedometer to keep track of physical activity was very high among Chinese Americans as they said they thought the pedometer helped increase their physical activity. For example, one participant stated: *“I looked at the pedometer every single day, as if it was a task for me to walk more and more everyday”.*

### Web-based intervention feasibility

Not surprisingly, lack of access to the internet, low computer skills/literacy, and low technology self-efficacy (especially among older adults) were identified as potential barriers precluding people from participating in online programs. *“The problem is, I don’t know how to get online,”* was a common sentiment shared by study participants. Furthermore, CAB members listed different learning styles and motivation levels among participants as factors that could affect the efficacy of the program. One shared that *“Some folks really enjoy learning new things…whereas other folks will be, like, ‘I am not going to do that,’ I will never be able to learn…”.*

### Web-based intervention implementation and modifications

Participants suggested the inclusion of more culturally appropriate foods and photos, the provision of videos and short explanations on the activities for better clarity, and formatting and translation of the contents in a way that is more familiar to the Chinese American community. There are some components that participants wanted in both English and Chinese but some components they thought should be kept solely in English. For example, participants said translating specialty foods, especially fruits and vegetables, to Chinese seemed unnecessary and sometimes confusing as they may have different names in Chinese due to different dialects. On top of that, participants preferred to keep the measurement unit in English, such as “oz” because that is how it appears on food labels. Keeping the unit in English would help them understand the portion size when they read the food labels.

In terms of online program implementation, both participants and CAB members agreed that providing technical support to participants is important and would increase the success of the online DPP. For example, *“If you want to put this (curriculum) online, you have to teach them how to use it. If they don’t know how and you ask them to do it, of course they will say it’s no good.”* CAB members also suggested using social media platforms that are popular with Chinese Americans, such as WeChat, to recruit potential study participants and to deliver the online program.

## Discussion

This study included community leaders, key informants, and stakeholders to investigate the potential barriers and feasibility of implementing a culturally and linguistically modified web-based DPP for Chinese Americans. Our findings shed some light on the barriers to behavioral change, acceptability, feasibility, and possible solutions for the translated DPP curriculum to be delivered online in the Chinese American community.

We found that comorbidities, perceived beliefs and practices, aging, lack of social support and family support hindered behavioral change. These barriers were also identified in other literature [27, 28]. Family support, especially, is a crucial factor that motivates participants to engage in healthy behaviors. For example, a qualitative study revealed that families are greatly involved in diabetes management among Chinese Americans with type 2 diabetes as a form of caring and enactment of responsibilities as family members [29]. In addition, perhaps due to the recommendations made by health care providers to remove refined carbohydrates from diets to control diabetes, Chinese participants often thought they have to eliminate staple foods such as rice and noodles when it comes to healthy lifestyle behavioral change [30]. This shows that the current practices do not emphasize the point that consumption of cultural foods should be controlled instead of eliminated; an important concept to incorporate in future DPP curriculum for Chinese Americans. The barriers and proposed solutions identified in this study could serve as a useful guide for developing web-based health promotion programs.

Lack of social support from health professionals appears to be an important barrier to health-related behavioral change. Some participants said that they received limited support from their doctors and were only given general information such as moving more and eating less, while they had no concrete ideas on how to achieve these goals and wanted more tactical guidance. A qualitative study suggested that Chinese patients are more passive and compliant in obtaining diabetes management information and tend to accept new knowledge unquestioningly when seeking medical advice [26]. It has been shown that general practitioners are perceived as important advisers and play an important role in promoting behavior change [31]. It is also suggested that doctors’ advice during a regular visit is the best way to motivate behavior change [32]. However, a national survey revealed that Asian Americans were less likely to receive counseling and less likely to report positive interactions with their doctors [33]. Similarly, Gleeson-Kerig [34] reported that many patients were dissatisfied with their doctors’ medical support as the physician focused more on the medical management issues vs. the prevention of these issues through lifestyle change, and patients felt judged by the physicians. There is often a gap between evidence-based clinical practice and real-world implementation [35]. Therefore, it is important for physicians to perform medical management and refer their patients to appropriate health programs at the same time during routine visits in order to overcome the barriers to behavioral change. Integration of social support should be considered in the web-based DPP to improve health outcomes.

Participants also provided feedback and suggestions on tailoring the curriculum content to be more culturally and linguistically appropriate. Some nutrition terms are unfamiliar to Chinese Americans’ daily life, such as “tonic and fitness water.” Moreover, the diet habits discussed in the curriculum were also different than their usual diet, which might prevent them from following the guidelines in the program. They suggested the diet and foods in the curriculum should be more related to Chinese or Asian diets. The pictures presented in the curriculum may also prevent them from adhering to the program as they see only Western faces instead of Chinese or Asians, and they would feel excluded from the program. Tabak et al. [36] indicated that the content adaptation of cultural and local foods could increase the program’s relevance. This shows that apart from changing the wording of food items, images with Chinese foods and faces are also as important for the participants. Wang et al. [20] showed that a culturally and linguistically sensitive curriculum could bring a positive effect on diabetes-related health outcomes. Thus, the content should be further adapted to create a feeling of inclusion. Focusing on cultural sensitivity is a critical aspect in health promotion programs and could increase the acceptance of the intervention [37].

Health strategies for healthy cooking, nutrition, physical activity and stress relief were also discussed. Some participants explored novel cooking methods such as using an air-fryer and blanching foods instead of frying and stir frying to reduce oil intake. Others suggested shopping with family as a way to prevent buying excessive unhealthy foods so they could remind each other. In terms of stress, some participants mentioned that stress induced an increase in fast food and sweets consumption, while others discussed doing exercises to reduce stress. From the healthy strategies proposed above, family and social support play an important role in behavior change among Chinese Americans. Some participants mentioned they have received support from friends and family by working out together to increase physical activity and sharing foods to reduce food intake. Jeffers et al. [38] suggested adding social support to the DPP curriculum to boost patients’ activation and increase the retention rate. However, the major social relations concern is that the participants are afraid to be treated or seen differently by others. Klasnja et al. [39] proposed that social platforms may have a negative impact on the participation rate. People who are not keeping up or who fall behind might feel stressed and afraid to share their activities and eventually drop out of the program [39]. Thus, social support may not be the primary tool due to social friction [39].

The acceptability of the translated PreventT2 curriculum was high. Participants reported they would be interested and would participate in future online curriculum. However, some participants mentioned that they would only engage in the health behaviors while taking the classes; once the program ends, they would eventually relapse and resume old habits. Therefore, reinforcement of healthy behaviors may take into consideration in future curricula. Jeffers et al. [38] emphasize the importance of reinforcement of change in diet and physical activity in the DPP curriculum. Evidence demonstrates habit formation process takes 2–3 months [40]. This suggests that future web-based interventions would benefit from a longer post-core intervention with positive reinforcement for successful program implementation.

Regarding web-based diet and physical activity self-monitoring tools, most participants thought the pedometers included in the DPP were useful in monitoring their physical activity. However, the acceptance of online diet monitoring tools varied among the participants depending on age and familiarity with technology. Participants who were more familiar with smartphone technology suggested taking photos of food they ate and upload them to social platforms as a means of diet monitoring as it is a common practice among people with smartphones. Tate et al. [41] proposed that the people who monitor their diet and physical activities may have greater behavior changes and weight reductions. Therefore, future intervention should consider collecting food photos from participants and analyze their behavioral changes pre- and post-intervention.

Despite the high acceptability of the in-person DPP curriculum, focus group participants conveyed mixed feelings about their ability to complete a web-based intervention. Some participants were concerned that the online DPP curriculum might not be feasible as most of them lacked internet access and/or knowledge of technology. Both study participants and CAB members cautioned that some Chinese Americans, especially seniors, might not have access to computers or smartphones; and even if they had access, they might not know how to use them. Sepah et al. also mentioned limited access to the internet might affect participant engagement and, thus, the overall efficacy of the online programs. [13] CAB members proposed that program staff work with senior centers and community health centers to provide internet access and technical support to study participants. In addition, using social media platforms that are familiar to community members may assist the participants in engagement and attendance as it allows them to interact with and help each other [42]. Moreover, there are currently multiple tools to help low literacy users. For instance, the built-in “speech-to-text” feature on smartphones allows users to write using a microphone and their own voice; or “text-to-speech” features to provide reading digital text aloud. Theses assistive technologies have been used in multiple education settings [43, 44], which may be adapted to web-based DPP interventions. Evidence has shown that curricula delivered with interactive support and feedback provides better health outcomes than self-guided interventions by reading materials alone [45].

As there is a need for rapid transition from in-person DPP curriculum to web-based curriculum due to the current coronavirus disease 2019 (COVID-19) pandemic, online delivery of translated DPP is essential for the Chinese American community to expand care delivery. Lee et al. believed that the online DPP curricula will become increasingly important, as more older adults will have computer access and obtain their health-related information online. [14] Sepah et al. updated the three-year follow-up after implementing the translated DPP curriculum online and found a long-term favorable result with body weight and HbA1c reduction. [46] Compared to an in-person DPP, online DPP intervention has a greater capacity for participant enrollment as an in-person DPP usually has limited seats and could be filled quickly [13]. The online DPP also offers greater flexibility in that participants can join and can read the education materials at any time that suits their schedules. Moin et al. [47] found that the participation and engagement rate was higher when the curriculum was delivered online.

There are limitations to this study. First, the majority of our participants came from the greater New York City metropolitan area. The findings may not be generalizable to people living in a more suburban or rural setting where lifestyle habits may differ. Also, within the Chinese population, Cantonese and Mandarin subgroups have both linguistic and cultural differences, and their dietary practices may differ. One should exercise caution when interpreting the study findings.

In conclusion, the acceptability of translated DPP in the Chinese American community in New York City is high. The major concern of the online DPP implementation from our focus group findings is the lack of internet access and limited technology knowledge. Social media platforms may help increase the efficacy of the program if online access and technical support is given. Thus, future studies with sufficient internet support with larger populations are needed to assess the efficacy of online DPP in the Chinese American community.

## Data Availability

The data that support the findings of this study are not openly available due to confidentiality regarding human subjects and are available from the corresponding author upon reasonable request.

## Abbreviations

CDC: Centers for Disease Control and Prevention
NYC: New York City
DPP: Diabetes Prevention Program
HbA1c: Hemoglobin A1c
CAB: Community advisory board
BMI: Body mass index
COVID-19: Coronavirus disease 2019

## References

[1] Divers J (2020). Trends in incidence of type 1 and type 2 diabetes among youths — selected counties and Indian Reservations, United States, 2002–2015. MMWR Morb Mortal Wkly Rep.

[2] Centers for Disease Control and Prevention (2020). National Diabetes Statistics Report 2020.

[3] Asian American Federation (2019). Profile of New York City’s Chinese Americans.

[4] Budiman A, Ruiz NG. Key facts about Asian Americans, a diverse and growing population. Pew Research Center. Published April 29, 2021. https://www.pewresearch.org/fact-tank/2021/04/29/key-facts-about-asian-americans/. Accessed 2 Nov 2021.

[5] Qin L, Knol MJ, Corpeleijn E, Stolk RP (2010). Does physical activity modify the risk of obesity for type 2 diabetes: a review of epidemiological data. Eur J Epidemiol.

[6] Fu M, Liu S, Lin Y-K, Chan W-Y (2018). Physical activity of Chinese American immigrants with type 2 diabetes/prediabetes: a mixed method study. Am J Nurs.

[7] Islam NS, Kwon SC, Wyatt LC (2015). Disparities in diabetes management in Asian Americans in New York City compared with other racial/ethnic minority groups. Am J Public Health.

[8] American Diabetes Association (2018). Economic costs of diabetes in the U.S. in 2017. Diabetes Care.

[9] Kim M, Berger D, Matte T. Diabetes in New York City: Public Health Burden and Disparities. New York: New York City Department of Health and Mental Hygiene, 2006.

[10] About the national diabetes prevention program. Centers for Disease Control and Prevention. https://www.cdc.gov/diabetes/prevention/about.htm. Accessed 7 June 2019.

[11] What Is the National DPP? Centers for Disease Control and Prevention. https://www.cdc.gov/diabetes/prevention/what-is-dpp.htm. Accessed 30 Oct 2021.

[12] Diabetes Prevention Program (DPP) Research Group (2002). The Diabetes Prevention Program (DPP): description of lifestyle intervention. Diabetes Care.

[13] Sepah SC, Jiang L, Peters AL (2014). Translating the diabetes prevention program into an online social network: validation against CDC standards. Diabetes Educ.

[14] Lee PG, Damschroder LJ, Holleman R, Moin T, Richardson CR (2018). Older adults and diabetes prevention programs in the veterans health administration. Diabetes Care.

[15] Wantland D, Portillo C, Holzemer W, Slaughter R, McGhee E (2004). The effectiveness of Web-based vs. non-Web-based interventions: a meta-analysis of behavioral change outcomes. J Med Internet Res..

[16] Torres-Ruiz M, Robinson-Ector K, Attinson D, Trotter J, Anise A, Clauser S (2018). A Portfolio Analysis of Culturally Tailored Trials to Address Health and Healthcare Disparities. Int J Environ Res Public Health..

[17] Young JJ (2014). Effectiveness of culturally tailored diabetes interventions for Asian immigrants to the United States: a systematic review. Diabetes Educ.

[18] Horowitz CR, Robinson M, Seifer S (2009). Community-based participatory research from the margin to the mainstream: are researchers prepared?. Circulation.

[19] Yeh MC, Heo M, Suchday S (2016). Translation of the diabetes prevention program for diabetes risk reduction in chinese immigrants in New York City. Diabet Med.

[20] Wang Y, Buchholz SW, Murphy M, Moss AM (2019). A diabetes screening and education program for Chinese American food service employees delivered in Chinese. Work Heal Saf.

[21] Tong A, Sainsbury P, Craig J (2007). Consolidated criteria for reporting qualitative research (COREQ): a 32-item checklist for interviews and focus groups. Int J Qual Heal Care.

[22] Prevent T2 Curricula and Handouts. Centers for Disease Control and Prevention. Published December 2, 2021. https://www.cdc.gov/diabetes/prevention/resources/curriculum.html. Accessed 3 May 2022.

[23] Braun V, Clarke V (2006). Using thematic analysis in psychology. Qual Res Psychol.

[24] Friese S. Qualitative data analysis with ATLAS.Ti – third edition. ATLAS.ti - The Qualitative Data Analysis & Research Software. Published May 12, 2022. https://atlasti.com/s-friese-qualitative-data-analysis-with-atlas-ti/. Accessed 30 Oct 2021.

[25] Dodgson JE (2019). Reflexivity in qualitative research. J Hum Lact.

[26] Choi TST, Walker KZ, Palermo C (2017). Culturally tailored diabetes education for chinese patients: a qualitative case study. J Transcult Nurs.

[27] Korkiakangas E, Taanila AM, Keinänen-Kiukaanniemi S (2011). Motivation to physical activity among adults with high risk of type 2 diabetes who participated in the Oulu substudy of the Finnish Diabetes Prevention Study. Health Soc Care Community.

[28] Whelan ME, Denton F, Bourne CLA (2021). A digital lifestyle behaviour change intervention for the prevention of type 2 diabetes: a qualitative study exploring intuitive engagement with real-time glucose and physical activity feedback. BMC Public Health.

[29] Chesla CA, Chun KM (2016). Accommodating type 2 diabetes in the Chinese American family. Qual Health Res..

[30] Chesla CA, Chun KM, Kwan CML (2009). Cultural and family challenges to managing type 2 diabetes in immigrant Chinese Americans. Diabetes Care.

[31] Keyworth C, Epton T, Goldthorpe J, Calam R, Armitage CJ (2020). Perceptions of receiving behaviour change interventions from GPs during routine consultations: a qualitative study. PLoS ONE.

[32] Mohamdy O (2021). Barriers to health behavior change in people with Type 2 diabetes: survey study. Int J Diabetes Clin Res.

[33] Ngo-Metzger Q, Legedza ATR, Phillips RS (2004). Asian Americans’ reports of their health care experiences: results of a national survey. J Gen Intern Med.

[34] Gleeson-Kreig J (2008). Social support and physical activity in type 2 diabetes: a social-ecologic approach. Diabetes Educ.

[35] Grimshaw J, Eccles M, Walker A, Thomas R (2002). Changing physicians’ behavior: what works and thoughts on getting more things to work. J Contin Educ Health Prof.

[36] Tabak RG, Sinclair KA, Baumann AA (2015). A review of diabetes prevention program translations: use of cultural adaptation and implementation research. Transl Behav Med.

[37] Resnicow K, Baranowski T, Ahluwalia JS, Braithwaite RL (1999). Cultural sensitivity in public health: defined and demystified. Ethn Dis.

[38] Skrine Jeffers K, Castellon-Lopez Y, Grotts J (2019). Diabetes prevention program attendance is associated with improved patient activation: Results from the Prediabetes Informed Decisions and Education (PRIDE) study. Prev Med reports.

[39] Klasnja P, Consolvo S, McDonald DW, Landay JA, Pratt W (2009). Using mobile & personal sensing technologies to support health behavior change in everyday life: lessons learned. AMIA Annu Symp proceedings AMIA Symp.

[40] Gardner B, Lally P, Wardle J (2012). Making health habitual: the psychology of ‘habit-formation’ and general practice. Br J Gen Pract.

[41] Tate DF, Wing RR, Winett RA (2001). Using internet technology to deliver a behavioral weight loss program. JAMA.

[42] Moin T, Damschroder LJ, Auyoung M (2018). Results from a trial of an online diabetes prevention program intervention. Am J Prev Med.

[43] Biancarosa G, Griffiths GG (2012). Technology tools to support reading in the digital age. Future Child..

[44] Wood SG, Moxley JH, Tighe EL, Wagner RK (2018). Does use of text-to-speech and related read-aloud tools improve reading comprehension for students with reading disabilities? a meta-analysis. J Learn Disabil.

[45] Willis EA, Szabo-Reed AN, Ptomey LT (2017). Do weight management interventions delivered by online social networks effectively improve body weight, body composition, and chronic disease risk factors? a systematic review. J Telemed Telecare.

[46] Sepah SC, Jiang L, Ellis RJ, Mcdermott K, Peters AL (2017). Engagement and outcomes in a digital diabetes prevention program: 3-year update. BMJ Open Diab Res Care..

[47] Moin T, Ertl K, Schneider J (2015). Women veterans’ experience with a web-based diabetes prevention program: a qualitative study to inform future practice. J Med Internet Res.

